# LFLDNet: Lightweight Fingerprint Liveness Detection Based on ResNet and Transformer

**DOI:** 10.3390/s23156854

**Published:** 2023-08-01

**Authors:** Kang Zhang, Shu Huang, Eryun Liu, Heng Zhao

**Affiliations:** 1 Engineering Research Centre of Molecular & Neuro Imaging of the Ministry of Education, School of Life Science and Technology, Xidian University, Xi’an 710071, China; 21121213323@stu.xidian.edu.cn (K.Z.); huangshu@uhan.net.cn (S.H.); 2 Zhejiang Provincial Key Laboratory of Information Network Technology, College of Information Science & Electronic Engineering, Zhejiang University, Hangzhou 310027, China; eryunliu@zju.edu.cn

**Keywords:** fingerprint liveness detection, spoofing attacks, lightweight, transformer, multi-head self-attention

## Abstract

With the rapid development of fingerprint recognition systems, fingerprint liveness detection is gradually becoming regarded as the main countermeasure to protect the fingerprint identification system from spoofing attacks. Convolutional neural networks have shown great potential in fingerprint liveness detection. However, the generalization ability of the deep network model for unknown materials, and the computational complexity of the network, need to be further improved. A new lightweight fingerprint liveness detection network is here proposed to distinguish fake fingerprints from real ones. The method includes mainly foreground extraction, fingerprint image blocking, style transfer based on CycleGan and an improved ResNet with multi-head self-attention mechanism. The proposed method can effectively extract ROI and obtain the end-to-end data structure, which increases the amount of data. For false fingerprints generated from unknown materials, the use of CycleGan network improves the model generalization ability. The introduction of Transformer with MHSA in the improved ResNet improves detection performance and reduces computing overhead. Experiments on the LivDet2011, LivDet2013 and LivDet2015 datasets showed that the proposed method achieves good results. For example, on the LivDet2015 dataset, our methods achieved an average classification error of 1.72 across all sensors, while significantly reducing network parameters, and the overall parameter number was only 0.83 M. At the same time, the experiment on small-area fingerprints yielded an accuracy of 95.27%.

## 1. Introduction

**Figure 1:**
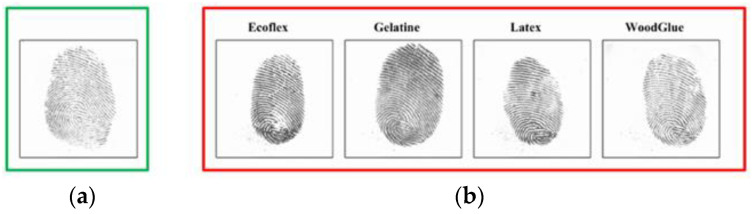
Samples of live (**a**) and spoofed fingerprints (**b**).

Automatic fingerprint identification systems (AFIS) have been widely used in personal identification and authentication for their high reliability, strong versatility and low cost. Due to the challenge of fingerprint spoofing attacks, the security of AFISs has received more and more attention. In practice, it is likely to be attacked by artificial fake fingerprints [1]. Artificial fingerprint replicas, also known as fake fingerprints, can be easily fabricated from a variety of inexpensive and commonly used materials, such as gelatine, silicone, wood glue, plasticine, etc. [2,3]. In addition, with the development of 3D printing technology, sophisticated 3D printing technology has been used for fingerprint spoofing attacks as well [4]. For example, a Brazilian doctor was arrested for using fake fingers made of silicone to deceive the biometric attendance system of a Sao Paulo hospital in March 2013. In March 2018, a gang in Rajasthan (India) bypassed the biometric attendance system by using wax-cast wood glue to forge fingerprints for providing proxies in the Police College Entrance Exam. Figure 1 shows some samples of live and spoofed fingerprints.

Hence, fingerprint liveness detection is of great significance for the further security of fingerprint applications. The various anti-spoofing approaches can be broadly classified into hardware-based and software-based methods [5]. Hardware-based methods need to utilize additional hardware devices to detect the characteristics of vitality, such as body temperature, humidity, blood pressure, pulse, blood oxygen saturation and so on [6]. Though external hardware devices can accurately differentiate between live and fake fingerprints, they also make the fingerprint recognition system more complex and expensive. What is more, it is hard to update these external hardware devices when attackers renew manufacturing techniques to improve hand-crafted fake fingerprints. Software-based solutions, on the other hand, extract features from the presented fingerprint image (or a sequence of frames) acquired by the fingerprint sensors, without incurring any additional hardware cost. Compared to hardware-based approaches, those that are software-based make fingerprint recognition systems low-cost and easy to update. Software-based methods are further divided into two types based on dynamic features (such as ridge deformation and sweating) and static features (such as ridge frequency, elastic characteristic skin, energy spectrum, etc.). Dynamic features are obtained from a time series of images, so the fingerprint acquisition process is very time-consuming. On the other hand, static features only need one or a few images for fingerprint liveness detection, which can not only prevent spoofing from attacking a fingerprint authentication system, but also is more convenient in practice.

A fingerprint liveness detection network with lightweight and high generalization based on ResNet and Transformer is proposed in this paper. The foreground of the fingerprint image is extracted by image processing methods such as adaptive thresholding, erosion and dilation to eliminate the influence of surrounding areas. Then, the image center point is chosen as the reference point for partitioning the image into local patches for training and testing of the network. This partition method is simple and effective, and it can generate small-area fingerprint images. More importantly, this method avoids directly extracting fingerprint minutiae and achieves an end-to-end effect. The combination of Residual Network and Transformer is used to construct a classification network architecture for fingerprint liveness detection, in which the 3 × 3 convolution kernels in the last block of the original Residual Network are replaced with MHSA blocks. Convolution can effectively learn abstract and low-resolution feature maps in large-scale images, and self-attention can process and summarize the detail information contained in feature maps. To improve the generalization ability of liveness detection on unknown materials, we used the style transfer CycleGan to fuse fingerprint images of different forged materials to simulate the generation of fake fingerprints of unknown materials.

This paper mainly aims to improve the generalization ability of the model for false fingerprints generated with different materials, and to reduce the computational burden with the goal of ensuring better fingerprint detection performance. The main contributions of this paper are as follows:

- End-to-end data structure. Different from most of the existing methods that randomly select fingerprint local patches, the fingerprint image center is used to guide the selection of local image patches to form the dataset, which can be used for direct training of the network without other transformations and avoiding the phenomenon of overfitting. The controllable patches partition does not change the size of the image and is an end-to-end operation that does not depend on minutiae. Moreover, the size of image patches can be adjusted to accommodate small-area fingerprint liveness detection.
- The improvement of network generalization ability. CycleGan is used to generate fingerprint images of unknown materials, expanding the dataset and improving the generalization ability of the network. The style migration can be carried out on the fingerprint image of known materials to generate a synthetic forged fingerprint image corresponding to unknown forged materials. From the LivDet2015 dataset, we can get two synthetic forged fingerprint images of ecoflex texture + gelatin style and gelatin texture + ecoflex style at the same time by training the pseudo fingerprint images of ecoflex and gelatin materials through CycleGan.
- Lightweight network structure. A new lightweight network architecture is proposed based on ResNet and Transformer. The network introduces the MHSA module in BotNet (a backbone network based on Transformer) and improves the residual structure in ResNet. The self-attention mechanism is introduced to integrate the global information on the high-level feature map, so that the network can pay more attention to the overall ridge structure characteristics of the fingerprint image. The proposed network structure greatly reduces the network depth and the number of convolution cores used in each layer, thereby effectively reducing the network parameters.

## 2. Related Works

Feature extraction is an important research topic in software-based liveness detection. The texture features of live fingerprints and fake ones differ in continuity, sharpness and ductility, so texture-based feature extraction methods have become the most commonly used methods among static feature-based methods.

Nikam [7] first proposed a method for extracting texture details based on LBP for fingerprint liveness detection. Some improvements of LBP, such as multi-scale local binary pattern [8] and unified local binary pattern [9], achieved high classification accuracy on some standard databases. The local phase quantization (LPQ) descriptor [10] obtained by short-time Fourier transform was proposed to distinguish real fingerprints from fake ones by using the information lost during the fabrication of fake fingerprints. In 2013, Gragnaniello et al. [11] exploited Weber local descriptors (WLD) to prevent spoofing attacks on fingerprint sensors. In 2015, Gragnaniello et al. [12] further proposed a new local contrastive phase descriptor (LCPD) which combined gradients with local phase information to achieve a commendable liveness detection accuracy. Xia et al. [13] proposed a new Weber local binary descriptor (WLBD) and evaluated the potential of the feature fusion method in the field of fingerprint liveness detection by analyzing different features and their aggregation methods.

These methods can be targeted at specific applications, and they have significant advantages such as rotation invariance and gray invariance. They can achieve high classification accuracy on some standard databases, but the overall generalization ability and robustness are unsatisfying, and it may be difficult to produce certain effects on images produced in some complex environments.

In addition to the handcrafted feature extraction mentioned above, more and more studies have used deep learning, such as MobileNet-v1, VGG-19, ResNet and GoogLeNet, to design highly robust and interpretable fingerprint liveness detection algorithms. Most of the existing methods based on CNN transfer the model pre-trained on natural images instead of redesigning a new network structure. However, there is a huge difference in complexity between fingerprint images and natural images, the network models are mostly prone to overfitting and they fail to achieve the expected effect on fingerprint liveness detection. Nogueira et al. [14] introduced a pre-trained VGG model for fingerprint liveness detection, which was significantly better than the previous algorithms in performance, achieved good results on the LivDet2011 and LivDet2013 datasets, and won the 2015 Fingerprint Liveness Detection Competition. Chugh, T. et al. [15] proposed a CNN-based method, which adopted a voting strategy based on multiple local patches centered on minutiae, showing state-of-the-art average classification accuracy. However, experiments on extracting fingerprint minutiae from fake fingerprint images have shown that their methods are unsatisfactory. The minutiae extracted from the fake fingerprint are not accurate, and more than 100 fingerprint minutiae can be extracted from many fake fingerprints, so the patches centered on each minutia greatly increase the computational cost and processing time, which is not suitable for real-time detection. Moreover, for the randomly cropped fingerprint image, adjusting the image resolution to adapt to the input size of the network will lead to the deformation of the fingerprint image, and the loss of some fingerprint information will lead to a decrease in classification accuracy. In the case of small-area fingerprints, the fingerprint minutiae may not be extracted, resulting in the failure of the method. Zhang et al. [16] modified the original residual network and named Slim-ResCNN, which was relatively light-weight but powerful and won the LivDet 2017 competition. In 2020, Zhang et al. [17] proposed FLDNet with only 0.48 M parameters.

At present, a large number of researchers have begun to use deep learning methods for fingerprint liveness detection. Such a method can save researchers a significant amount of manual design on the algorithm, and the model can learn autonomously from the data. Eliminating many preprocessing and intermediate steps, the learned model has a certain generalization ability, and many studies have shown that deep learning methods have certain advantages in performance on fingerprint live detection tasks, with indicators leading most traditional algorithms, lower average detection time consumption and better overall performance.

## 3. Fingerprint Foreground Segmentation and Patches Extraction

The pre-processing of fingerprint images includes two steps: foreground segmentation and local patches extraction. In the whole fingerprint image, the surrounding area of the fingerprint does not contain any useful information and is removed by foreground segmentation, and then the center of the fingerprint image is used to locate local patches from the foreground area, which reduces the network execution time and model parameters. The fingerprint image foreground segmentation mainly includes the following steps:

### 3.1. Adaptive Threshold Segmentation

**Figure 2:**
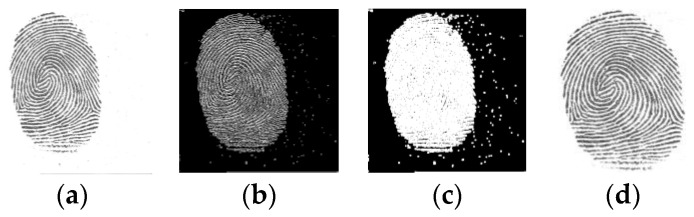
Fingerprint foreground extraction: (**a**) original fingerprint, (**b**) fingerprint segmented with adaptive thresholding, (**c**) dilated fingerprint and (**d**) fingerprint after foreground extraction.

According to the gray distribution of pixels in different regions of the image, local thresholds are adaptively calculated by a local mean or Gaussian weighted average for foreground or background segmentation of each pixel. In this paper, the segmentation threshold of each pixel is determined by the 3 × 3 neighborhood mean. Given $T_{p}=Mean-Delta$, where $T_{p}$ is the local threshold, Mean represents the 3 × 3 neighborhood average of pixels and Delta is an adjustment offset, set to −2. The effect of setting the adjustment offset Delta here is that, for the background area, the local threshold can be increased, thereby forcing the background grayscale to 0. The binarized image segmentation is performed on each pixel using the local threshold of the pixel. The original fingerprint image and the segmented fingerprint image are shown in Figure 2a,b, respectively.

### 3.2. Dilation and Connected Components with Stats

For the fingerprint image segmented by adaptive threshold, we expect the fingerprint area to be as separate from the background as possible, so we use the dilation method to expand the highlighted white part of the image. Due to the noise interference of the original image, there may be small white patches around the fingerprint in the image after the above two processes, so the largest white block is selected as a more accurate fingerprint area. Figure 2 shows the process of fingerprint foreground extraction.

**Figure 3:**
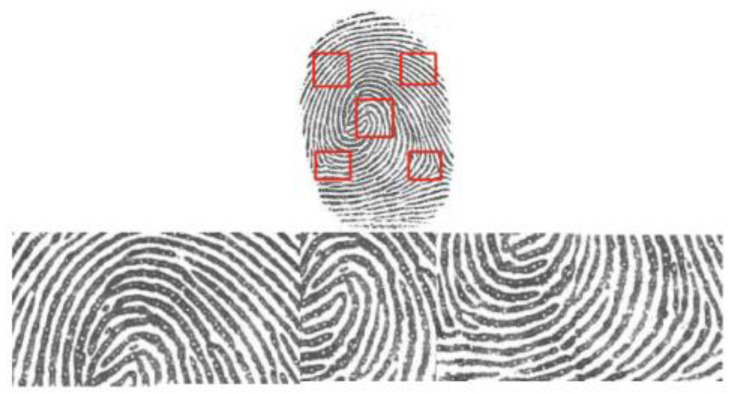
Multiple local patches extracted from the foreground of fingerprint image.

After foreground extraction, to unify the size of the fingerprint image, we partition a local patch of w × w (w = 112) from the foreground region of the fingerprint in the center of the fingerprint image. We also select four points at the upper left, lower left, upper right and lower right in steps of 56 pixels of the center point. Then, we cut a w × w patch centered on each of these four points. This blocking method takes full advantage of the entire fingerprint image information. However, some of these selected patches might include little fingerprint information, which is not conducive to training the network. Hence, they must be excluded from the training set. Color reversal and normalization are performed on each w × w local patch, and then the maximum closure of the binary patch is obtained. When the maximum closure area of local patch is more than 60% of the local patch area, it will be selected as one sample, otherwise it will be excluded. Furthermore, the extracted local patches will be rotated at four different angles, 0°, 90°, 180° and 270°, to deal with the problem of insufficient fingerprint samples, as shown in Figure 3.

## 4. Network Structure

**Figure 4:**
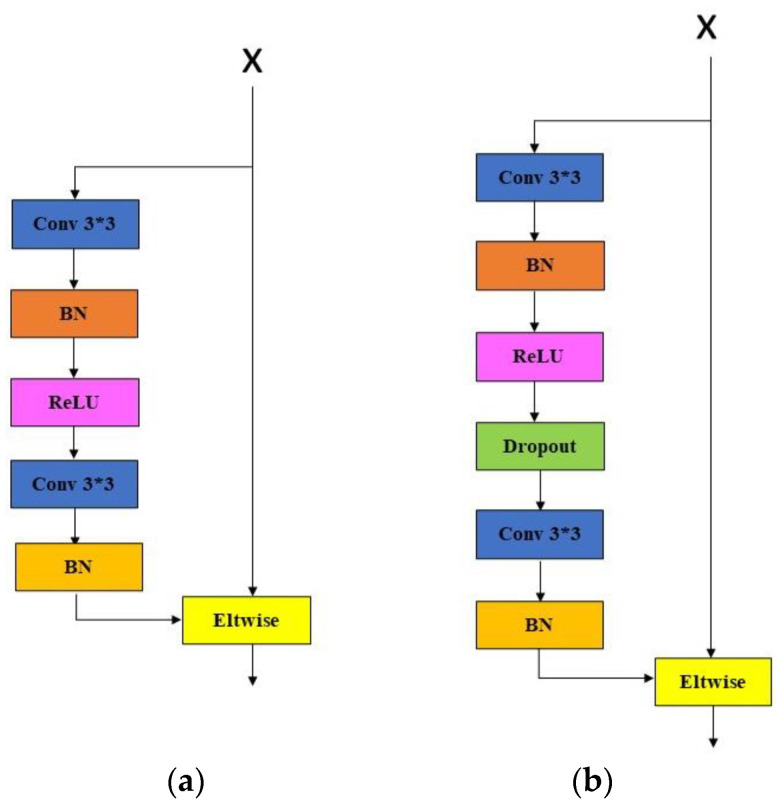
The original residual blocks (**a**) and the improved residual blocks (**b**).

Most of the existing fingerprint liveness detection networks use the residual network as the skeleton and make modifications based on this. However, it is easy to cause network overfitting in small databases, especially on grayscale images with relatively simple structural features such as fingerprints, which is not directly suitable for fingerprint liveness detection tasks. We propose a lightweight fingerprint liveness detection network based on ResNet and Transformer. In terms of residual blocks, we borrowed some ideas from the Slim-ResCNN network [16] and a dropout layer was added to each residual block to reduce network overfitting. The modified residual structure is shown in Figure 4. We believe that the texture information of fingerprints is particularly important in fingerprint liveness detection, especially the continuity and structural shape of the ridges, so we introduced the self-attention mechanism in the Transformer into the network to make the network pay more attention to the overall ridge relationship of the fingerprint image. CNN effectively learns abstract information in large-scale images through convolution and obtains low-resolution feature maps, while self-attention layers can summarize and process high-level semantic information contained in low-resolution feature maps to improve network performance and computational efficiency.

**Figure 5:**
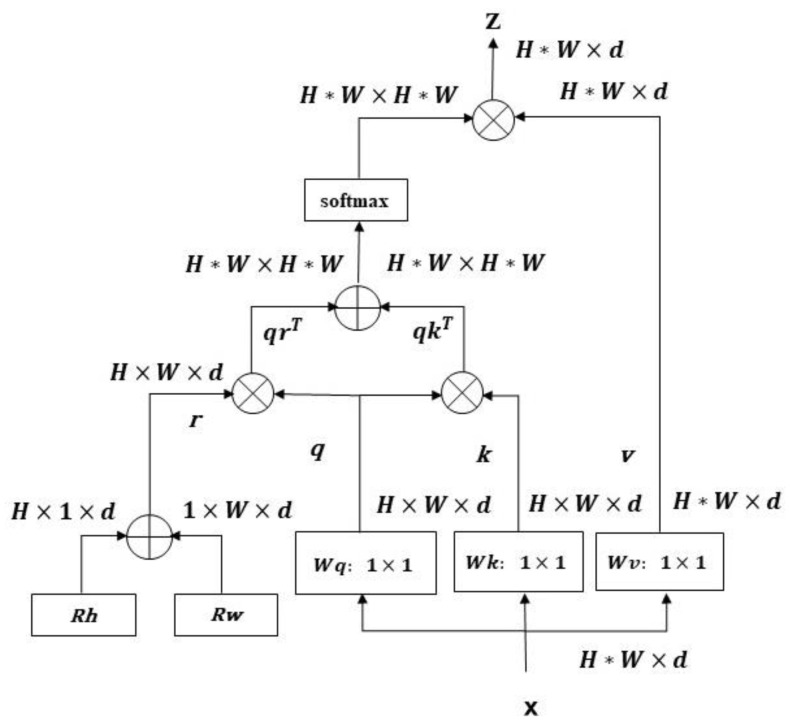
Structure diagram of multi-head self-attention layer.

The multi-head self-attention layer is shown in Figure 5. The dimension of the input feature map is represented as H×W×d, and three 1×1 trainable parameters of $W_{q}$, $W_{k}$ and $W_{v}$ are used to convolve the feature map to obtain three matrices, the q query matrix, k key matrix and v value matrix, with the size being H×W×d. Unlike applications in NLP tasks, where positional encoding is done before word vectors enter the network, it is not easy to do positional encoding before input in CV tasks, so two learnable vectors Rh and Rw were embedded in the MHSA used, and two matrices with dimensions H×d and W×d are obtained, and then expanded to H×1×d and 1×W×d; the two are added to get the matrix r, which is regarded as the spatial attention of the horizontal and vertical dimensions to complete the position encoding. Then, r and q are multiplied to get the relationship between content and location ${qr}^{T}$, and q and k are multiplied to get the content-to-content query ${qk}^{T}$. The similarity feature obtained by adding content–position and content–content is multiplied by v after softmax, so that MHSA can focus on the appropriate area. We used four MHSAs to project features into multiple subspaces to improve the expressiveness of the model.

**Figure 6:**
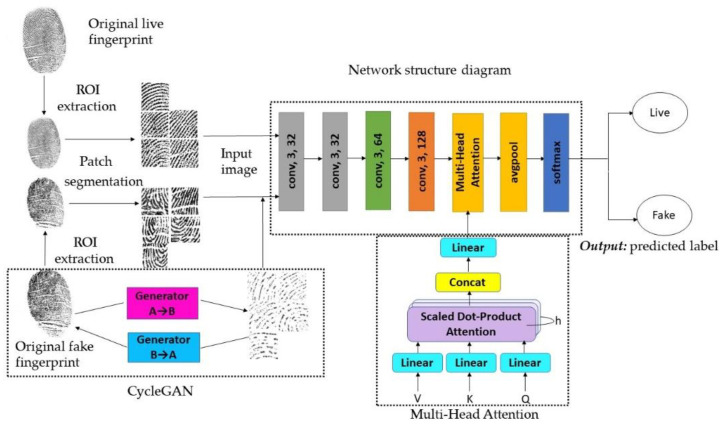
The overall framework of the proposed fingerprint liveness detection network.

Following the principles of neural network structure design, a binary classification network structure was constructed for spoof presentation attacks detection. The proposed network consists of seven parts: Conv1, Conv2, Conv3, Conv4, Conv5, global average pooling layer and final classification layer. The overall framework of the proposed fingerprint liveness detection network is shown in Figure 6. In order to make the network can be more lightweight, we greatly reduced the depth of the network and the number of convolution kernels used in each layer; for example, there are only 32 convolution kernels in the Conv1 layer.

The structure of the network is shown in Table 1, and the network structure is explained as follows: (1) Conv1 is responsible for connecting the input local patches and extracting the initial features passed to the subsequent residual blocks. The image input size of the modified network is required to be 112 × 112. (2) Conv1 is followed by Conv2, Conv3 and Conv4. Deeper image feature information is extracted through convolution operations, and the size of the output feature map in some layers will be halved. We doubled the number of convolution kernels to ensure that the total amount of learnable parameters remains unchanged, as can be seen from Conv3 and Conv4. (3) In Conv5, the MHSA module is used to replace the original 3 × 3 convolution kernel. (4) To reduce network model parameters, the global average pooling layer is used instead of the fully connected layer. (5) The network is trained on local patches using a cross-entropy loss function. The network structure is shown in Table 1. Compared with the image size used in object detection and segmentation (such as 1024 × 1024), in image classification tasks we often deal with relatively small image sizes, such that VGG network and ResNet originally required image input size of 224 × 224, This will cause the feature map of the original ResNet to only have a size of 7 × 7 when entering the last layer of bottleneck, which is not conducive to the processing of global feature information by the last self-attention layer. Therefore, in our proposed network, the input size of the image was modified to 112 × 112, which is just in line with the local patch size after preprocessing, and the size of the feature map when entering the Conv5 block was enlarged to 28 × 28 compared to the original network. The stride in the MHSA layer in the Conv5 block was uniformly changed to 1 to increase the feature map resolution and thus improve the accuracy of the final classification.

By referring to the relevant literature, and based on our own experiments, we obtained the optimized relevant parameters for the size of the image being 112 × 112. We trained our models using stochastic gradient descent with batch size 32 for 200,000 iterations. The initial learning rate was set to 0.01, initial momentum was 0.9 and it was reduced by 20% per 50,000 iterations. A dropout layer was added after each convolutional layer (except the first one) and the dropout rates were set to 0.2.

## 5. Fingerprint Image Style Transfer

One of the major limitations of current spoof detection methods is their unsatisfying generalization performance across “unknown” or novel spoof materials that were not used during training of the spoof detector. It has been shown that the selection of spoof materials used in training (known spoofs) directly impacts the performance against unknown spoofs. In particular, Chugh and Jain [18,19] analyzed the material characteristics (two optical and two physical) of 12 different spoof materials to identify a representative set of 6 materials that cover most of the spoof feature space. With the increasing popularity of fingerprint authentication systems, hackers are constantly devising new fabrication techniques and novel materials to attack them. As a result, it is not feasible to include all potential spoof fabrication materials in training a spoof detector. Sandouka and Bazi, Y. [20,21] used a network with EfficientNets as the backbone and a GAN network for generating additional images to solve the problem of the poor generalization ability of fingerprint PAD across sensors and compared with other GAN networks and non-GAN networks.

We use a CycleGAN-based [22] style transfer method to improve the cross-material generalization performance of fingerprint liveness detectors.X and Y represent two different datasets, and the model needs to train two mappings: G:X→Y and F:Y→X. Two discriminators, Dx and Dy, have been introduced; Dx is used to determine whether the image comes from x or F(y), and Dy is used to determine whether the image comes from y or F(x). For cross-material scenarios, we hypothesized that the style information from fake fingerprint images of known material can be transferred to synthesize fake fingerprint images that can be made from unknown materials, which could improve the model’s performance for novel materials while preserving its performance on known materials.

The loss function of CycleGAN mainly includes three parts: adversarial loss function, cycle-consistent loss function and identity loss function. The fingerprint image has many details in structure, such as minutiae, ridges and pores, etc. The role of the adversarial loss function is mainly to make the generated image more realistic. The cycle-consistent loss function ensures that the generated image retains the content part of X and only changes the style part of it. Identity loss function maintains the original fingerprint features during the migration process, preventing the generator from adjusting the features autonomously and changing the overall image. The loss function is shown in Equations (1)–(5):

{(1)LGANG,DY,X,Y=Ey~Pdata(y)log⁡DY(y)+Ex~Pdata(x)log⁡(1−DYGx)(2)LGANF,Dx,Y,X=Ex~Pdata(x)log⁡DX(x)+Ey~Pdata(y)log⁡(1−DXGy)

Equations (1) and (2) refer to the cross-entropy loss function, and they are dual relations. Taking Equation (1) as an example, like the loss function of the conventional GAN network, it is a process of mutual game between D and G, where D tries to distinguish between synthetic images and real images, while G tries to cheat D by generating realistic images. The goal of G is to minimize this objective function, while the goal of D is to maximize the objective function. The optimal solution is when $P_{x\sim data(x)}=P_{y\sim data(y)}$.

During the training process, our sample x obtains a fake image y through the generator, which tends to become a value that can deceive the discriminator. The generator will gradually discover that no matter what x is sent in, as long as the output of the generator is more similar to y, it can deceive the discriminator. To retain the content of x and only change the internal style, we introduce a cycle-consistent loss function to solve this problem, as shown in Equation (3):

(3) $$L_{cycle}\left(G,F\right)=E_{x\sim P_{data(x)}}\left[{\left\|F(G\left(x\right))-x\right\|}_{1}\right]+E_{y\sim P_{data(y)}}{\left\|G(F\left(y\right))-y\right\|}_{1}$$

The cyclic consistency loss adopts L1 loss, in order to constrain $\hat{x}$ = $F\left(G\left(x\right)\right)$ = x, so that the $G\left(x\right)$ generated by G can still be consistent with x in content. Similarly, for y, the cyclic consistency loss can prevent the generator from deceiving the discriminator by ignoring the input image x and only changing the style part of the generated image to ensure that the generated image retains the content part of x. In addition, identity loss is also used in CycleGAN, as shown in Equation (4):

(4) $$L_{Identity}\left(G,F\right)=E_{y\sim P_{data(y)}}\left[{\left\|G\left(y\right)-y\right\|}_{1}\right]+E_{x\sim P_{data(x)}}{\left\|F\left(x\right)-x)\right\|}_{1}$$

The total loss function of the network is shown in Equation (5):

(5) $$L_{loss}=L_{GAN}\left(G,D_{Y},X,Y\right)+L_{GAN}\left(F,D_{x},Y,X\right)+{\lambda_{1}L}_{cycle}\left(G,F\right)+\lambda_{2}L_{Identity}\left(G,F\right)$$

**Figure 7:**
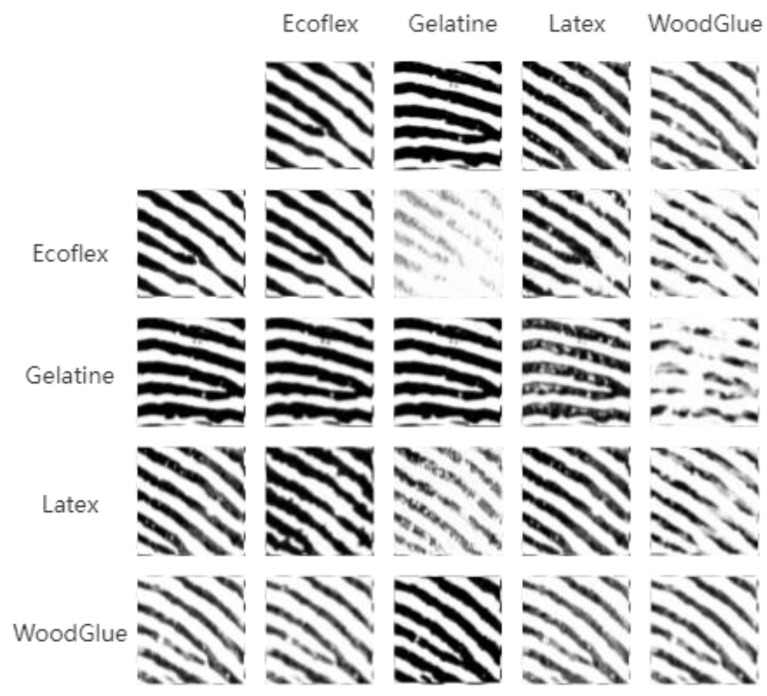
CycleGan style transfer to generate fingerprint images with different styles.

The weight ratio of adversarial loss function, cycle-consistent loss function and authentication loss function is set to 1:10:10. During training, the batch size is set to 1, the number of training rounds is 100, the Adam optimizer is used, the initial learning rate is set to 0.0002, the learning rate is fixed for the first 50 rounds, and the learning rate is decayed to 0 in equal parts for the last 50 rounds. The result of CycleGan style transfer is shown in Figure 7.

The leftmost column of Figure 7 represents false fingerprints from known materials, and the top row of Figure 7 provides different style elements. Through style transfer, the fingerprint images of the leftmost column will generate false fingerprint images with corresponding style according to the fingerprint images of the top row.

## 6. Datasets

The following datasets were used in this research:

LivDet Dataset: To evaluate the performance of the proposed method, we used the LivDet 2011 [23], LivDet 2013 [24] and LivDet 2015 [25] datasets. Each of these datasets contained more than 16,000 fingerprint images acquired from four different fingerprint readers. The CrossMatch and Swipe readers in the LivDet 2013 dataset were not included in the research since the fingerprint data of the CrossMatch reader was abnormal, which is discouraged for evaluation. The resolution of the fingerprint image obtained by the Swipe reader was only 96 dpi, which was different from other LivDet datasets. In LivDet 2015, the test set consisted of fake fingerprints fabricated from new and unknown materials in the training set. These new materials included Liquid Ecoflex and RTV for the Biometrika, Digital Persona and GreenBit readers, and OOMOO and gelatin for the CrossMatch reader. Table 2 summarizes the LivDet datasets included in the research.

## 7. Performance Evaluation Metrics

In all the experiments for this paper, we followed the metrics used in LivDet:

Classification accuracy (Accuracy) was defined as the ratio of the number of samples correctly classified by the classifier to the total number of samples for a given test dataset. $F_{errlive}$ was the percentage of misclassified live fingerprints. $F_{errfake}$ was the percentage of misclassified spoof fingerprints. The last of these was the average classification error rate (ACE) [15,16].

The output of the Softmax layer of the trained model was in the range [0–1], and we averaged all patch outputs of the fingerprint to get the score. The threshold for determining the liveness of fingerprints was set to 0.5. The fingerprint image with a liveness score over 0.5 was considered as an “alive” entity, otherwise it was considered as a “spoof” artifact. 

## 8. Experimental Results and Analysis

The proposed method was tested in the following two scenarios to evaluate the effectiveness of the algorithm.

Known Sensors and Known Materials Scenario: In this case, all images were captured using the same sensor for training and testing, and all the materials used to make the deceptions in the test set were known. The ACE of the proposed network was compared with that of several existing works. The results are shown in Table 3.

Most of the current SOTAs in LivDet2011 and LivDet2013 used MobileNet-V1 for training and testing on local patches based on fingerprint minutiae. The method proposed in this paper showed a slight improvement for the data of the Digital reader and the Italdata reader in the LivDet 2011 dataset and for the data of the Italdata reader in the LivDet 2013 dataset, and the overall average accuracy rate was not far from the best result. The method of [15] needs to extract minutiae from the fingerprint image, and then segment the fingerprint image based on the minutiae. However, it is not easy to extract minutiae points on fake fingerprints. When we extract the minutiae points of fake fingerprints through Verifinger, we find that there will be many wrong minutiae and the average number of minutiae extracted is more than 80, which will divide a fingerprint image into more than 80 local patches. During testing, all local patches need to be tested and scored, and then a weighted average obtained, which is not suitable for real-time fingerprint liveness detection systems. In comparison, the local fingerprint partition method proposed in this paper is simpler, and there are only 5 fingerprint local patches at most from a fingerprint image. For testing the same fingerprint image, the time consumption can be shortened twelvefold, and the final accuracy is almost the same.

Known Sensor and Unknown Material Scenario: In this case, the fingerprint images in the training and test sets were captured by the same sensor. But new materials that were not known during training were used in the test set. The detailed performance comparison between the proposed algorithm and other algorithms is shown in Table 4. When all the spoof fabrication materials were known during the training, the metric was referred to as $F_{errfakeknown}$, and for cases where all the spoof fabrication materials to be encountered during testing were not known during training, the metric was referred to as $F_{errfakeunknown}$.

The network model obtained by the fingerprint image training enhanced by style transfer had a significant improvement in the recognition accuracy for forged fingerprints of unknown materials. The $F_{errfakeunknown}$ indicators for the Biometrika, CrossMatch, Digital Persona and GreenBit readers were improved from 2.60, 1.34, 3.40 and 2.60 to 1.20, 0.00, 2.60 and 1.40. At the same time, the style transfer did not affect the recognition and judgment of the model for known material fingerprints and real fingerprints, which can be seen from the two indicators of $F_{errfakeknown}$ and $F_{errlive}$. Compared with the other algorithms, it can be found that our proposed method showed a significant improvement compared with the methods of the LivDet2015 and LivDet2017 competition champions [16]. Compared with the method proposed in [15], our method also had a slight advantage in the recognition accuracy for unknown materials. It can be shown that the style transfer of fingerprint images via CycleGAN can enhance the generalization ability of the network model to fake fingerprints of unknown materials. Moreover, we list the comparison with the ACE of some other experimental methods in Table 5 and the accuracy on the LivDet 2015 datasets in Table 6.

In addition, in order to verify that the method proposed in this paper can solve the problem of liveness detection for small-area fingerprints, we also tested the accuracy of small-area fingerprint recognition on the LivDet2015 database, and the results are shown in Table 7.

Because there were no related references for small-area fingerprint liveness detection, this paper did not make a comparison with other works. In the test results for small-area fingerprints, the results of the data for the Digital Persona reader were poor, and the overall ACE was 7.27. What was more abnormal was the data tested on the CrossMatch reader. After the fusion of five local patches, a high recognition accuracy could be obtained, but the results of the test on a single small-area fingerprint were not particularly good. The results for the data in the GreenBit reader were the best, with an overall ACE of 3.18 and an overall average ACE of 4.73.

Since the running speed of the network model will be affected by many factors such as the experimental environment and the amount of input data each time, we demonstrated the superiority of the network by comparing the sizes of network parameters. We compared the parameters of the network proposed in this paper with those of the Slim-ResCNN used by the champion of the 2017 fingerprint liveness detection competition and those of the commonly used lightweight networks MobileNet-V1 and MobileNet-V2. The network used in [15] was MobileNet-V1, and its parameters were 4.04 M. The parameters of MobileNet-V2 were 2.19 M. The parameter number for Slim-ResCNN was 2.15 M. The parameter number for the network proposed in this paper was only 0.83 M. It can be seen that the number of network parameters proposed in this paper was significantly smaller than those for the other networks. Table 8 compares the sizes of network parameters.

**Figure 8:**
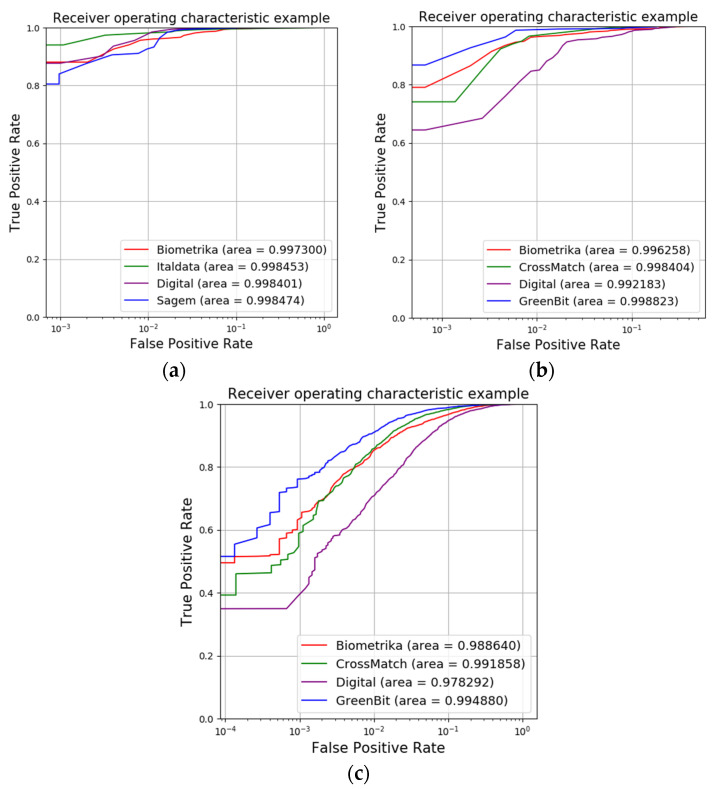
ROC curves: (**a**) LivDet2011, (**b**) LivDet2015 and (**c**) LivDet2015 small-area fingerprint.

We drew the ROC curves for the small-area fingerprint recognition results of LivDet2011, LivDet2015 and LivDet2015, and these are shown in Figure 8. In order to facilitate viewing, the ROC curves of LivDet2011 and LivDet2015 are enlarged in the 0–0.2 parts.

## 9. Conclusions

This work studied the problem of fingerprint liveness detection and proposed a simple and effective method for fingerprint foreground and local patch extraction. Furthermore, we designed a lightweight fingerprint liveness detection network based on a multi-head self-attention mechanism, and we enhanced the generalization ability of the model through style transfer. By modifying the convolutional neural network and adding the MHSA mechanism to improve recognition accuracy, we significantly reduced the network parameters. The CycleGAN network was used for the style transfer of fingerprint images, enhancing the generalization ability of the fingerprint live detection system. The proposed method achieved excellent results on the LivDet2011, LivDet2013, and LivDet2015 datasets; on the LivDet 2015 dataset, our methods achieved an average classification error of 1.72 across all sensors, outperforming most of the state-of-the-art methods, while significantly reducing network parameters, with the overall parameter number only 0.83 M. At the same time, the experiment on small-area fingerprints yielded an accuracy of 95.27%. In the future, we will explore more effective FLD technology, further improve the accuracy of fingerprint liveness detection on the LivDet datasets and explore other GAN-based models to improve the cross-sensor generalization ability between different sensors to deal with fingerprint deception attacks.

## Figures and Tables

**Table 1:** Lightweight network structure.

Layer	Output	Kernel
Conv 1	112 × 112	3 × 3 × 32
Conv 2	112 × 112	3 × 3 × 32 3 × 3 × 32
Conv 3	56 × 56	3 × 3 × 64 3 × 3 × 64
Conv 4	28 × 28	3 × 3 × 128 3 × 3 × 128
Conv 5	28 × 28	$\left(\begin{array}{c} 1\times 1\times 128\\ MHSA\\ 1\times 1\times 128 \end{array}\right)\times 2$
Avg pool	1 × 1	-

**Table 2:** LivDet datasets information.

Dataset	Sensor	Image Size	Live Image (Train/Test)	Fake Image (Train/Test)	Spoof Materials
LivDet 2011	Biometrika	315 × 372	1000/1000	1000/1000	Ecoflex, Gelatin, Latex, Silgum, Wood Glue
	ItalData	640 × 480	1000/1000	1000/1000	
	Digital Persona	355 × 391	1000/1000	1000/1000	Gelatin, Latex, PlayDoh, Silicone, Wood Glue
	Sagem	352 × 384	1000/1000	1000/1000	
LivDet 2013	Biometrika	315 × 372	1000/1000	1000/1000	Ecoflex, Gelatin, Latex, Modasil, Wood Glue
	ItalData	640 × 480	1000/1000	1000/1000	
LivDet 2015	GreenBit	500 × 500	1000/1000	1000/1500	Ecoflex, Gelatin, Latex, Wood Glue, Liquid Ecoflex, RTV
	Biometrika	1000 × 1000	1000/1000	1000/1500	
	Digital Persona	252 × 324	1000/1000	1000/1500	BodyDouble, Ecoflex, PlayDoh, OOMOO, Gelatin
	CrossMatch	640 × 480	1510/1500	1473/1448	

**Table 3:** LivDet2011 and LivDet2013 results (%).

Dataset	Sensor	State-of-the-Art	VGG	AlexNet	Proposed
LivDet 2011	Biometrika	1.24 [15]	5.2	5.6	2.55
	Digital	1.61 [15]	3.2	4.6	1.15
	Italdata	2.45	8.0	9.1	1.55
	Sagem	1.23 [26]	1.7	3.1	1.52
	Average	1.63	4.53	5.6	1.69
LivDet 2013	Biometrika	0.20 [15]	1.8	1.9	0.75
	Italdata	0.30 [15]	0.4	0.5	0.20
	Average	0.25	1.1	1.2	0.48

**Table 4:** LivDet2015 results (%).

Method	LiveDet2015	$F_{errlive}$	$F_{errfake}$	$F_{errfakeknown}$	$F_{errfakeunknown}$	Ace
LivDet 2015-winner [27]	Biometrika	8.50	3.73	2.70	5.80	5.64
	CrossMatch	0.93	2.90	2.12	4.02	1.90
	Digital Persona	8.10	5.07	4.60	6.00	6.28
	GreenBit	3.50	5.33	4.30	7.40	4.60
	Average	4.78	4.27	3.48	5.72	4.49
SlimResCNN [16]	Biometrika	3.55	4.23	2.44	7.72	3.10
	CrossMatch	1.72	3.91	3.18	4.58	4.32
	Digital Persona	4.28	4.78	3.73	6.40	2.37
	GreenBit	2.22	2.65	2.42	3.11	2.64
	Average	2.94	3.89	2.94	5.45	3.11
FLDNet [17]	Biometrika	1.01	3.67	2.61	5.75	2.34
	CrossMatch	0.87	2.21	**0.69**	4.45	1.54
	Digital Persona	3.26	1.90	1.43	2.85	2.58
	GreenBit	0.71	0.41	0.52	**0.21**	0.56
	Average	1.46	2.05	1.31	3.31	1.76
Fingerprint Spoof Buster [15]	Biometrika	**0.90**	1.27	**0.60**	2.60	1.12
	CrossMatch	**0.80**	**0.48**	0.82	**0.00**	0.64
	Digital Persona	**1.97**	**1.17**	**0.85**	1.80	1.48
	GreenBit	**0.50**	0.80	0.30	1.80	0.68
	Average	**1.02**	**0.93**	0.64	1.48	0.97
Proposed Method	Biometrika	3.70	2.07	1.80	2.60	2.72
	CrossMatch	1.67	0.96	0.71	1.34	1.32
	Digital Persona	5.20	2.47	2.00	3.40	3.56
	GreenBit	1.70	1.13	0.40	2.60	1.36
	Average	2.91	1.66	1.24	2.44	2.20
Proposed Method + CycleGAN	Biometrika	3.70	**0.87**	0.70	**1.20**	2.00
	CrossMatch	1.00	0.82	1.41	**0.00**	0.91
	Digital Persona	4.80	2.20	2.00	2.60	3.24
	GreenBit	0.90	**0.60**	**0.20**	1.40	0.72
	Average	2.6	1.12	1.08	1.73	1.72

The best performance results are highlighted in bold.

**Table 5:** ACE comparisons with existing methods on LivDet 2015 datasets (%).

Method	Green Bit	Biometrika	Digital Persona	Crossmatch	Average
WLBD [13]	4.53	13.72	10.82	9.94	9.68
FPAD [28]	1.2	3.2	**2.28**	4.6	2.82
DRN [29]	4.77	6.24	6.8	3.46	5.32
VGG-19 [14]	4.6	5.6	6.3	1.9	4.6
SlimRes-CNN [16]	2.64	3.10	2.37	4.32	3.11
FLDNet [17]	**0.56**	2.34	2.58	1.54	1.76
Proposed + CycleGAN	0.72	**2.0**	3.24	**0.91**	**1.72**

The best performance results are highlighted in bold.

**Table 6:** The accuracy on the LivDet 2015 datasets (%).

Datasets	Proposed + CycleGAN
GreenBit	98.54
Biometrika	97.23
Digital Persona	95.11
Crossmatch	98.01
Average	97.22

**Table 7:** LivDet2015 small-area fingerprint experiment results (%).

LiveDet2015	$F_{errlive}$	$F_{errfake}$	$F_{errfakeknown}$	$F_{errfakeunknown}$	Ace
Biometrika	6.12	3.10	2.11	5.09	4.30
CrossMatch	3.74	4.79	6.67	2.77	4.26
Digital Persona	9.69	5.73	5.07	7.06	7.27
GreenBit	3.32	3.09	1.62	6.01	3.18
Average	5.50	4.17	3.77	5.14	4.73

**Table 8:** Comparison of network parameters and test time.

	Proposed	Slim-ResCNN	MobileNet-V1	MobileNet-V2
Parameters	0.83 M	2.15 M	4.04 M	2.19 M
Time	0.07132 s	0.196 s	0.5389 s	0.226 s

## Data Availability

The data presented in this study are available on https://livdet.org/.
